# Medical students’ experiences learning intimate physical examination skills: a qualitative study

**DOI:** 10.1186/1472-6920-14-39

**Published:** 2014-02-28

**Authors:** Andra M Dabson, Parker J Magin, Gaynor Heading, Dimity Pond

**Affiliations:** 1 School of Medicine and Population Health, University of Newcastle, University Dr, Callaghan, NSW 2308, Australia; 2 School of Medicine and Population Health, University of Newcastle, University Dr, Callaghan, NSW 2308, Australia; 3 GP Training Valley-to Coast, Gavey St, Mayfield West, NSW 2308, Australia; 4 Health Education and Training Institute, Victoria Rd, Gladesville, NSW 2111, Australia; 5 School of Medicine and Population Health, University of Newcastle, University Dr, Callaghan, NSW 2308, Australia

**Keywords:** Medical student, Intimate physical examination, Learning experiences, Teaching techniques

## Abstract

**Background:**

Intimate physical examination skills are essential skills for any medical graduate to have mastered to an appropriate level for the safety of his or her future patients. Medical schools are entrusted with the complex task of teaching and assessing these skills for their students. The objectives of this study were to explore a range of medical students’ experiences of learning intimate physical examination skills and to explore their perceptions of factors which impede or promote the learning of these skills.

**Methods:**

Individual semi-structured interviews (N = 16) were conducted with medical students in years two to five from the University of Newcastle, as part of a larger research project investigating how medical students develop their attitudes to gender and health. This was a self-selected sample of the entire cohort who were all invited to participate. A thematic analysis of the transcribed data was performed.

**Results:**

Students reported differing levels of discomfort with their learning experiences in the area of intimate physical examination and differing beliefs about the helpfulness of these experiences. The factors associated with levels of discomfort and the helpfulness of the experience for learning were: satisfaction with teaching techniques, dealing with an uncomfortable situation and perceived individual characteristics in both the patients and the students. The examination causing the greatest reported discomfort was the female pelvic examination by male students.

**Conclusions:**

Student discomfort with the experience of learning intimate physical examination skills may be common and has ongoing repercussions for students and patients. Recommendations are made of ways to modify teaching technique to more closely match students’ perceived needs.

## Background

Teaching physical examination to medical students could be assumed to be a fairly straightforward task. Students need a systematic approach, a good knowledge of what are normal findings, and a method of describing and analysing their findings – a blend of knowledge, technical and cognitive skills. This process is, however, greatly complicated by the fact that physical examination is an interaction between two (or sometimes more) individual human beings, each bringing to the interaction his or her own knowledge, experience, beliefs, attitudes, status and cultural context [1]. Each person is potentially open to harm from the interaction. Nowhere are these layers of complexity more obvious than in the physical examination of genitalia and breasts.

Intimate examination (physical examination of genitalia, the female breast and rectal examination) is generally taught in medical schools either intentionally or opportunistically using a number of strategies: didactic lectures or demonstrations, individual practice on inanimate simulation (such as mannequins or part-trainers), individual practice with simulated patient/trainers, or individual practice on patients in a clinical setting with varying degrees of supervision [2]. Some analyses of these methods of teaching have been made in terms of how effectively students have learned the skills taught and to some extent how comfortable students were with the experience [3-6]. Mostly the evidence is collected in terms of self-report of efficacy and comfort, though some were formally assessed [3].

At the University of Newcastle (the setting for the study reported in this paper) the years of study are not specifically designated as “pre-clinical” and “clinical”. Physical examination skills are explicitly taught, beginning in the second semester of the first of five years of study. The students are initially introduced to the general physical examination and are subsequently taught a detailed technique of examination for each system during the period in which they study medical and clinical sciences and relating to particular clinical disciplines. Intimate examination is usually begun in clinical terms in the third year and is opportunistic, depending on which patients are seen on clinical placements. Formal teaching of the pelvic examination is done during the obstetrics and gynaecology rotation in fourth year. Volunteer clinical teaching associates (who act as teacher/patients, allowing and assisting students to examine them) assist students for a session during the obstetrics and gynaecology rotation. Some students may have earlier ad hoc exposure to these examinations during clinical attachments in general practice in their first three years of study.

Standard texts of physical examination generally address the interpersonal aspect of the examination with a brief paragraph on the need for students to consider the patient’s concerns and fears, but most do not consider the possibility that a student might have concerns and fears [7,8], though these are relatively common [9]. Bates Guide to Physical Examination and History Taking [10] states that a student *“may feel uneasy or uncomfortable …”* about examining a patient but discusses this very briefly and in terms of the student needing to master this for the good of the patient: *“Helping the patient relax is essential for an adequate examination”.*

Our objectives (within a broader study of medical students, gender and health) were, firstly, to explore a range of medical students’ experiences of learning intimate physical examination skills in the curriculum and their personal responses to those experiences. Secondly, we sought to explore the students’ perceptions of the factors which impeded or promoted their learning experiences.

## Method

This paper presents and considers the implications of one of the core themes of a larger research project into how medical students develop their attitudes to the interaction between gender and health.

### Participants and recruitment

An invitation to express interest in participation was emailed to all undergraduate (years 1–5) medical students in the Faculty of Health in the University of Newcastle. Subsequent choice of students to invite to interviews was to have been purposive, reflecting maximum variation sampling on the basis of sex, year of course, age, ethnic and cultural background.

### Study design

All participants were interviewed individually face-to-face (by AD). The interviews were semi-structured, recorded (with consent) on audiotape, transcribed, and the transcription returned to the individual interviewee for review. While an interview schedule informed discussions, interviews were informant-led as far as possible and concurrent data collection and analysis led to iterative modifications of the interview schedule [11]. The interview question relating to general physical examination and intimate physical examination was: *“Tell me about learning physical examination”*. Probes were: *“Has your gender or the patient’s gender affected this?”* and *“What about intimate physical examination?”*. Data collection was to continue until thematic saturation was achieved.

### Data analysis

Transcriptions were analysed using an inductive coding approach [12], aided by the use of N6 software. A thematic analysis was performed and themes linked and grouped to develop a schema for interpreting the data [13]. A representative sample of the data was independently coded by two researchers (AD and PM) and minor differences in interpretation resolved by consensus.

The transcript of each interview was returned to the participant to ensure it was accurate and reflected the opinions of the participant. The participant was asked to correct or annotate the transcript and return it; all changes were accepted. Reflexivity was maintained by the two researchers involved in the data analysis being cognisant throughout of their own personal context as, respectively, female and male practising clinicians and educators, and of any potential effect this may have had on their interpretation of the data.

### Ethics

Clearance for the research was obtained from the University of Newcastle Human Research Ethics Committee. Signed consent was obtained from each participant.

## Results

Only eighteen students responded to the study invitation, making maximum variation sampling impractical. Instead, respondents were interviewed sequentially. After 16 interviews, thematic saturation had been achieved, both for the overall study aim of exploring experiences of gender in the course and for the emergent theme of intimate physical examination presented in this paper, and data collection ceased.

Despite the lack of purposive sampling, the resultant respondent population included students from years two to five of the medical course, aged 20 to 32, and of both sexes (Table 1). The group also included domestic, international and Aboriginal and Torres Strait Islander students as well as participants who self-identified as homosexual or bisexual (details of individual participants’ background and ethnicity are not provided in this paper in order to maintain their anonymity).

**Table 1:** Demographics of participants

**Interview**	**Year of course**	**Sex**	**Age group**
1	2	F	20-22
2	2	M	23-26
3	3	F	23-26
4	2	F	20-22
5	3	F	23-26
6	4	F	20-22
7	4	F	20-22
8	4	F	20-22
9	4	F	20-22
10	4	M	23-26
11	5	M	23-26
12	5	F	23-26
13	5	F	27+
14	3	M	27+
15	4	F	20-22
16	3	F	20-22

The medical course at the University of Newcastle involves clinical patient contact from the first semester of first year, and all the participants had experience with physical examination and intimate examinations to a greater or lesser degree.

The interviews lasted one to one and a half hours.

A recurring topic raised by the participants in the context of gender and medical education was the issue of physical examination and, in particular, intimate physical examination. This was brought up spontaneously by several of the early participants and because of the inductive nature of the research this led to subsequent participants being specifically asked about their experiences with physical examination and prompted about intimate physical examination. All of the participants spoke about learning intimate physical examination and many of the participants expressed dissatisfaction and/or discomfort with their experiences when learning these skills.

Participants’ views about their experiences learning intimate physical examination were consistent with three dominant sub-themes: satisfaction with teaching techniques, dealing with an uncomfortable situation and individual characteristics.

### Satisfaction with teaching techniques

There was much variability in participants’ level of satisfaction with their tuition in intimate physical examination ranging from “mostly well done” *(interview 10: male, year 4, age 23–26)* to “very poor” *(interview 16: female, year 3, age 20–22).*

Apart from a single structured tutorial on pelvic examination involving clinical teaching associates, participants stated that the teaching of intimate physical examination was sporadic and opportunistic.

Most of the participants thought that the structured tutorial on pelvic examination was a good idea, though perceptions of its value as a learning experience were very mixed. Some female participants thought that the tutorial was really useful, but described their male colleagues as being very uncomfortable with it. Male participants described it as “weird” and “surreal” and spoke in sometimes contradictory ways about it. They appeared torn between seeing it as a valuable, supportive learning experience and a very confusing personal experience:

“I thought it was fantastic learning experience but then again … I haven’t done one since, so I don’t know … I couldn’t even remember to tell you how I did it or what the technique or what I’m looking for, so I don’t know that it’s necessarily a … brilliant teaching experience, or good for your career …” (interview 10: male, year 4, age 23–26)

If the teaching of pelvic examination received mixed reviews, the teaching of other intimate examination skills appeared to the participants to be non-existent. A participant who had performed his first rectal examination during a general practice placement believed that his general practice supervisor assumed he had been taught this skill in the curriculum:

“Rectal exam that I did first off … wasn’t taught all that well by the GP I was with. There was no teaching. … I’ve received no teaching at all from the faculty”. (interview 14: male, year 3, age 27+)

A number of participants said that on most occasions they had little idea of what they should be doing or looking for in intimate physical examinations, and stated that they hoped that by the time they had done a number of examinations that this would become clearer for them. Some commented that tutors often seemed to have “forgotten what it’s like not to know”, or assumed that students had already been taught what to look for and what their findings meant.

Most of the participants stated that the majority of their learning of the interpersonal aspects of the technique of physical examination came from watching their tutors in the clinical setting. They described seeing intimate physical examination done by tutors. Some examinations they thought were comfortable and appropriate for the patient, some where the examination was appropriate though the tutor and patient appeared uncomfortable, and some that involved poor technique or disrespect for the patient:

“… probably the biggest way that we’ll be taught by the clinicians … that’s sort of the bedside teaching, that’s what you see, and how well they do that really affects how you will treat your patients”. (interview 15: female, year 4, age 20–22)

### Dealing with an uncomfortable situation

All the participants found the learning of any physical examination difficult to some extent, particularly as they felt that the examinations were not for the benefit of the patients, but only for the students’ learning purposes. This led to reluctance on the part of participants to approach patients to ask permission to examine them, even when the examination was not an intimate examination. Individual participants seemed quite conflicted by the apparently opposing needs of their own education and the patients’ rights to humane and respectful treatment.

“*… you sort of abridge your examination to try and make it a little better, so you don’t have to put the patient through as much prodding. And that’s particularly so as a student, because you know you’re just doing it for your learning, you’re not doing it for their treatment”. (interview 12: female, year 5, age 23–26)*

Intimate physical examination was an even more complex issue, and none of the participants would consider approaching a patient about an intimate examination. They relied on their supervisors to broach the possibility with the patient and to negotiate what part in the examination the student would play.

After the Pap smear was taken and [the supervisor] performed a vaginal exam, she also said, “Do you mind if [the participant] practices?” And it was … so much better. I still felt very uncomfortable because … I don’t feel comfortable in those situations, but I persevered with it because I think I need to know this stuff. (interview 14, male, year 3, 27+)

Female participants felt that while they were uncomfortable about learning intimate physical examination, their male colleagues generally found the process of learning more difficult than they did, particularly for pelvic examinations. Participants described some of their male colleagues’ reactions this way:

No one wants to learn a rectal exam. That’s uniform, male, female, the same. The boys revolted themselves I think a lot by having to do the female vaginal exam. (interview 12: female, year 5, 23–26)

Most of the boys are pretty disgusted by it really. So they did their first ever … but they didn’t have to do a Pap smear or anything just a vaginal exam, and were pretty disgusted by it. (interview 5: female, year 3, age 23–26)

The methods they described to deal with their discomfort related to intimate examinations, involved avoidance of the situation or modifications of their own behaviour, the methods being more or less adaptive in terms of coping.

#### Avoidance

The situation was avoided either by not approaching the patients at all or by standing back and just watching and denying the necessity of personal experience as a student. They felt that they would learn it more quickly and more appropriately when they were doctors. In the context of an intimate physical examination a participant commented:

“I haven’t been able to examine many patients. I don’t think that’s a negative thing. I think that medicine you just learn by seeing patients come through and seeing problems and it’s the best to learn, but you don’t necessarily have to examine them”. (interview 10: male, year 4, age 23–26)

#### Behaviour modification

“Managing” the situation of an intimate physical examination generally involved one or more of three main methods: “getting it over with”, “being really professional” and stress management techniques.

##### “Getting it over with”

A number described doing the examination “as quickly as possible”, even leaving out some of the examination techniques, to reduce their own and/or the patient’s discomfort. This often involved trying not to think about the patient as a person:

They just do it, because they have to do it, and a friend of mine said he just gritted his teeth, went in and did it and walked out, so he tried not to personalise himself with the patient too much. It was … like he just tried to walk in and say, “OK, I’m just doing the vaginal examination”, and walk out and not chat to the patient too much and get to know them. That’s how he sort of disassociated himself. (interview 6: female, year 4, age 20–22)

##### Being “really professional”

Some would adopt a very “professional” role which involved dressing appropriately, being very serious with the patients and offering very “professional” explanations for what they were doing. They described this technique as a method to make the patient more comfortable, but the tone of the descriptions implied that there were personal benefits in the technique in terms of dealing with anxiety for the student as well.

So I guess it’s all very serious, you get in there, you need to check this, and if they are unsure about things, like sometimes you get the sense they’re a bit hesitant, then you explain, “Well checking for hernias is part of the gastro-intestinal examination and I have to go through it systematically”, and most of the time they are fine with it and it’s quick. (interview 15: female, year 4, 20–22)

##### Stress management

Less adaptive stress management techniques were also described, such as joking about the patients in a sexual manner, or drinking alcohol to cope with feelings about the experience of examining a patient. Sexual joking was seen largely as a method used by male students, and a participant *(interview 1: female, year 2, age 20–22)* described her discomfort when male colleagues described having examined the “hottest” patient during a clinical attachment.

A number of participants described discussions about intimate examination that occurred between students in the context of drinking “at the pub”, though only one male participant commented directly on using alcohol to manage his feelings about an intimate examination:

We had the tutorial where we had to practice breast and vaginal examinations and it was one of most surreal experiences I’ve ever had, and I think I got very drunk the night before and very drunk the night after just trying to cope with what had just happened in my life. (interview 10, male, year 4, 23–26)

### Individual characteristics

Specific individual characteristics in both the students and the patients were believed to make the physical examination in general, and intimate physical examination in particular, more or less difficult.

#### Patient factors

The participants felt that they were more comfortable examining older patients than younger patients. A participant *(interview 10: male, year 4, age 26)* stated that, “I don’t know whether it’s just a personal thing or not, but a similar examination on a younger person would be less comfortable.” He felt that this may have been because an older person would be more willing to be examined, or perhaps less apparently sexual.

Participants felt that it was easier to examine patients from rural areas, who were perceived to be more “relaxed” about it.

Participants of both sexes felt that it was easier to examine a patient of their own sex.

And in terms of physical examination, I’ve really had no problems with the female patients, and with male patients it’s just an awareness that they will be embarrassed about some things. (interview 15: female, year 4, age 20–22)

#### Student factors

Participants of both sexes also commented that intimate physical examination was easier for female students, for older students with more life-experience and for students with “common sense”. The cultural background of the students also had an impact on students’ coping styles. Participants from cultures with even stronger taboos against interpersonal physical contact than are present in Anglo-Australian culture were felt by the participant him or herself and by their colleagues to be more likely to avoid practising all their physical examination skills as well as intimate physical examination skills.

I don’t want to do obstetrics and gynaecology, because I don’t feel comfortable with [doing pelvic examinations] … and it’s probably because of my traditional background, my cultural background … I don’t feel comfortable working with, or dealing with women’s problems. (interview 14, male, year 3, 27+)

## Discussion

Given the strength of the cultural rules about physical contact present in even a seemingly relaxed Western culture [14,15], it might be *expected* that students learning physical examination techniques would find performing physical examination uncomfortable [16]. This would be even more so for learning the skills of genital, breast and rectal examination [17-19]. There is little overt recognition of this difficulty in medical culture in general, or in medical training in particular. Therefore students are often left unaided to deal with the difficulties they encounter but may not understand at a conscious level. In the last few years some authors have recognised this difficulty [4-6,20-22], though follow-through into curricular change does not appear to have occurred generally. Indeed, Nensi et al’s survey of Canadian medical schools published in 2012 found that there was very little teaching time and less assessment of the skill of digital rectal examination in their courses [23].

A “sink or swim” approach to teaching important skills has a venerable history in medical education [24], and indeed most students ultimately do manage, but there can be significant costs. Many students learn intimate physical examination skills imperfectly and often only finally in the setting of internship, where at least they are “doing it *for* the patient”, not for themselves [25]. Unsupported experience can lead students who find these skills particularly uncomfortable to acquire to change their career options or more often continue to hide their lack of skills well into their working lives [26,27].

There is an expectation that the responsibility for how well a consultation goes lies firmly with the doctor [28], and by implication (and often explicit instruction) with the medical student. This adds to the emotional burden of inexperienced trainees just learning their skills, complicating an already difficult task.

We suggest that overtly acknowledging the inherent difficulties in physical examination and addressing the interpersonal aspect of examination as well as the physical techniques may reduce the stress of many of the students, not just of those who may really struggle with the process otherwise. This could assist the students to be, and feel, more competent in performing the skills. It may also produce graduates who are better at handling the concerns and expectations their patients may bring to physical examination. The difficulty of teaching both the technical and interpersonal skills of physical examination has already been recognised to some extent in the use of clinical teaching associates in Australia and overseas for undergraduate and postgraduate teaching [29,30]. Mathewlynn et al. [31] and Siwe et al. [22] have suggested that clinical teaching associates are particularly helpful for male students who have been found to be more anxious about learning pelvic examination skills [20,32]. While Powell et al. [33] found that both male and female students were able to perform fewer examinations on the opposite sex during their training and were less confident with these examinations, the finding that the greatest distress around this issue was described as being in male students learning to perform pelvic examinations is not described elsewhere.

Recognition in medical schools that learning intimate physical examination is uncomfortable for many students [6] and a particular difficulty for some because of their cultural backgrounds or their individual personal skills and coping styles, can, and has in some cases, lead to further changes in the way physical examination is taught. Training in physical examination skills can be done initially on inanimate simulation models [5] and with expert supervision so that the students can address the motor skills and understanding of findings first and then progress to deal with these in a clinical setting when they are more competent [3,6,34]. The use of volunteer teacher/patients or clinical teaching associates at this stage in the training would appear appropriate [6,35], though Seago et al. have found better results where the clinical teaching associate was utilised prior to further simulation training [32]. Some flexibility in progression from training on simulation models to clinical situations should be included because of the differences in individual student’s skills and experience.

It may be useful to explicitly acknowledge the importance of role-modelling in acquiring intimate physical examination skills, and to offer training to tutors and lecturers in role-modelling skills and teaching in front of patients. This teaching methodology would be consistent with aspects of learning that our participants found helpful.

### Limitations of this study

This small qualitative study was undertaken in a single medical school in a particular cultural setting and thus its findings may not be able to be generalised. The issue of learning intimate physical examination was raised spontaneously by a number of participants and all of the participants had found this difficult. There is recognition elsewhere [4,6] that learning these skills is problematic, which does make it likely that this issue is not isolated to this institution.

The limited number of participants may not completely reflect the breadth of student views on this topic, though those participants did represent a variety of sex, age group, training level and cultural background.

### Suggestions for further research

A quantitative study of a larger sample of students across several medical schools would clarify the proportion of students who have difficulties with learning intimate physical examination and allow measurement of the level of distress related to this issue, and the student and teaching methodology factors associated with the distress.

## Conclusion

This study revealed that emotional discomfort with the learning of physical examination in general, and in intimate physical examination in particular, may be common among the medical students in this school and perhaps more generally. The intensity of this response appears to vary depending on individual characteristics of the student, the clinical situations the students experience and the teaching techniques used by their clinical supervisors.

High levels of discomfort do affect the students’ learning and mastering of important clinical skills.

Recognition of the complexity of these skills and adapting teaching techniques to address this may improve the clinical skills of graduates and reduce the stress of medical students.

## Competing interests

The authors declare that they have no competing interests.

## Authors’ contributions

AD conceived of the study, participated in its design and co-ordination, performed the interviews, participated in the data analysis and drafted the manuscript. PM participated in the design of the study and the data analysis. GH participated in the design of the study and the data analysis. DP participated in the design and co-ordination of the study. All authors read and approved the final manuscript.

## Authors’ information

AD performed this research as part of a research higher degree. PM, GH and DP were the candidate’s supervisors.

## Pre-publication history

The pre-publication history for this paper can be accessed here:

http://www.biomedcentral.com/1472-6920/14/39/prepub
